# 3-Carbamoyl-1-(2-nitrobenzyl)pyridin­ium bromide

**DOI:** 10.1107/S1600536812015917

**Published:** 2012-04-18

**Authors:** Kyung Beom Kim, Kwang-Deog Jung, Cheal Kim, Youngmee Kim

**Affiliations:** a Department of Fine Chemistry, Seoul National University of Science & Technology, Seoul 139-743, Republic of Korea; b Clean Energy Research Center, Korea Institute of Science & Technology, Seoul 130-650, Republic of Korea; c Department of Chemistry and Nano Science, Ewha Womans University, Seoul 120-750, Republic of Korea

## Abstract

In the title compound, C_13_H_12_N_3_O_3_^+^·Br^−^, the benzene and pyridinium rings form a dihedral angle of 82.0 (1)°. In the crystal, N—H⋯Br and N—H⋯O hydrogen bonds link the components into chains along [001]. In addition, weak C—H⋯O and C—H⋯Br hydrogen bonds are observed.

## Related literature

The title compound was prepared as an NAD^+^ (nicotinamide adenine dinucleotide) model. For effective regeneration systems for co-enzymes (*e.g.* NADH), see: Hollmann *et al.* (2001 ▶); Lee *et al.* (2011 ▶); Maenaka *et al.* (2012 ▶); Park *et al.* (2008 ▶); Ruppert *et al.* (1988 ▶); Zhu *et al.* (2006 ▶). For the mechanisms of redox inter­conversions (NADH/NAD^+^), see: Zhu *et al.* (2003 ▶); Song *et al.* (2008 ▶).
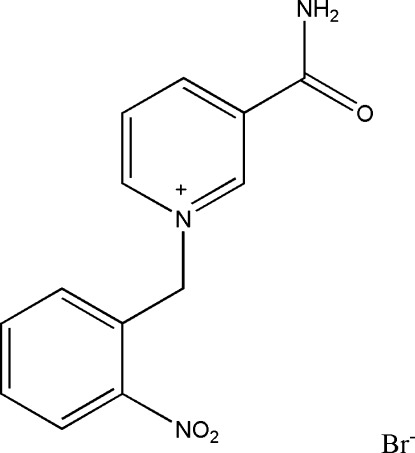


## Experimental

### 

#### Crystal data

C_13_H_12_N_3_O_3_^+^·Br^−^
*M* *_r_* = 338.17
Monoclinic, 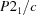

*a* = 17.576 (4) Å
*b* = 7.9990 (16) Å
*c* = 10.152 (2) Å
β = 105.88 (3)°
*V* = 1372.8 (5) Å^3^
*Z* = 4
Mo *K*α radiation
μ = 3.01 mm^−1^
*T* = 293 K
0.15 × 0.15 × 0.10 mm

#### Data collection

Bruker SMART CCD diffractometer
Absorption correction: multi-scan (*SADABS*; Bruker, 1997 ▶) *T*_min_ = 0.661, *T*_max_ = 0.753
7399 measured reflections
2684 independent reflections
2081 reflections with *I* > 2σ(*I*)
*R*_int_ = 0.034

#### Refinement

*R*[*F* ^2^ > 2σ(*F* ^2^)] = 0.036
*wR*(*F* ^2^) = 0.086
*S* = 1.04
2684 reflections
187 parameters
2 restraints
H atoms treated by a mixture of independent and constrained refinement
Δρ_max_ = 0.36 e Å^−3^
Δρ_min_ = −0.38 e Å^−3^

### 

Data collection: *SMART* (Bruker, 1997 ▶); cell refinement: *SAINT* (Bruker, 1997 ▶); data reduction: *SAINT*; program(s) used to solve structure: *SHELXS97* (Sheldrick, 2008 ▶); program(s) used to refine structure: *SHELXL97* (Sheldrick, 2008 ▶); molecular graphics: *SHELXTL* (Sheldrick, 2008 ▶); software used to prepare material for publication: *SHELXTL*.

## Supplementary Material

Crystal structure: contains datablock(s) I, global. DOI: 10.1107/S1600536812015917/lh5450sup1.cif

Structure factors: contains datablock(s) I. DOI: 10.1107/S1600536812015917/lh5450Isup2.hkl

Supplementary material file. DOI: 10.1107/S1600536812015917/lh5450Isup3.cml

Additional supplementary materials:  crystallographic information; 3D view; checkCIF report

## Figures and Tables

**Table 1:** Hydrogen-bond geometry (Å, °)

*D*—H⋯*A*	*D*—H	H⋯*A*	*D*⋯*A*	*D*—H⋯*A*
N1—H1*B*⋯O1^i^	0.86 (1)	2.30 (1)	3.143 (4)	168 (4)
N1—H1*A*⋯Br1^ii^	0.86 (1)	2.61 (1)	3.454 (3)	166 (3)
C4—H4⋯Br1	0.93	2.82	3.743 (3)	173
C7—H7*B*⋯Br1^iii^	0.97	2.82	3.595 (3)	137
C5—H5⋯O2^iv^	0.93	2.36	3.271 (4)	167
C3—H3⋯O1^i^	0.93	2.27	3.150 (4)	157

Symmetry codes: (i) 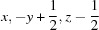; (ii) 
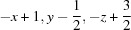
; (iii) 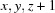; (iv) 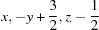.

**Fig. 1.**
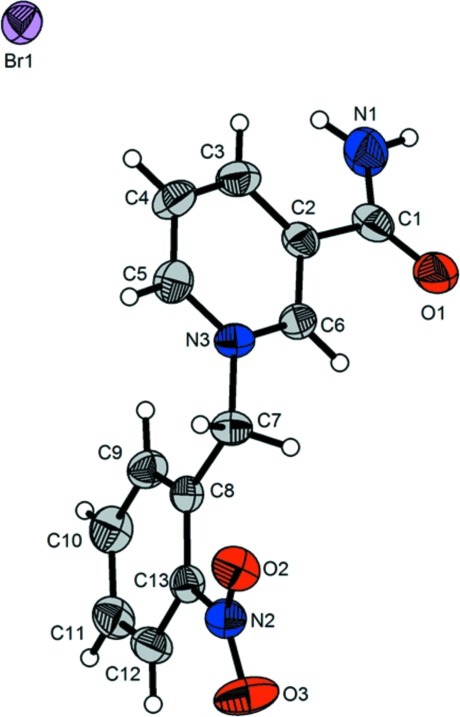
The molecular structure with displacement ellipsoids shown at the 50% probability level.

## supplementary crystallographic information

### Comment

One of the most important challenges in applying mono-oxygenases reactions in vitro is to find an effective regeneration system for the necessary co-enzyme (mostly NAD(P)H) (Hollmann *et al.*, 2001; Lee *et al.*,2011; Maenaka *et al.*, 2012; Park *et al.*, 2008; Ruppert *et al.*, 1988; Zhu *et al.*, 2006). The well established methods for the regeneration of the nicotinamide co-enzyme mainly consist of an enzyme-coupled approach utilizing formate dehydrogenase or glucose-6-phosphate dehydrogenase. Because the redox coenzyme couple NADH/NAD^+^ is ubiquitous and controls so much of our oxidation/reduction nature, there has been a long-standing interest in the mechanisms of the redox interconversions (Zhu *et al.*, 2003). The high cost of these co-factors, however, is prohibitive of industrialization of many promising enzymatic processes. An efficient method of their *in situ* regeneration is the only means for making the processes economically and industrially feasible (Song *et al.*, 2008). Therefore, many researchers have given considerable attention to the chemistry of NADH and its models (Hollmann *et al.*, 2001). In this work, we have synthesized the title compound as a NAD^+^ model and report herein its crystal structure.

The molecular structure of the title compound is shown in Fig. 1. The benzene ring (C8-C13) and pyridine ring (N3/C2-C6) form a dihedral angle of 82.0 (1)°. In the crystal, intermolecular N—H···Br and N—H···O hydrogen bonds link the components to form chains along [001]. In addition, weak C—H···O and C—H···Br hydrogen bonds are observed.

### Experimental

Nicotinamide (123.4 mg, 1 mmol) was dissolved in 10 ml acetonitrile. After stirring for a few minutes, 2-nitrobenzyl bromide (220.4 mg, 1 mmol) was carefully added to the reaction mixture. The solution was stirred for 3 h at 353K. The precipitate was filtered, washed three times with methylene chloride, and dried under vacuum. Crystals suitable for X-ray analysis were obtained from a methanol soution of the title compound in a few days.

### Refinement

H atoms bonded to C atoms were placed in calculated positions with C—H distances of 0.93 Å for aromatic C atoms and 0.97 Å for a methylene C atoms. They were included in the refinement in riding-motion approximation with *U*_iso_(H) = 1.2*U*_eq_(C). The positions of N—H atoms of the amine were refined with N—H = 0.860 (2) Å and *U*_iso_(H) =1.2*U*_eq_(N).

### Crystal data

**Table:**

C_13_H_12_N_3_O_3_^+^·Br^−^	*F*(000) = 680
*M**_r_* = 338.17	*D*_x_ = 1.636 Mg m^−^^3^
Monoclinic, *P*2_1_/*c*	Mo *K*α radiation, λ = 0.71073 Å
Hall symbol: -P 2ybc	Cell parameters from 11909 reflections
*a* = 17.576 (4) Å	θ = 2.7–27.6°
*b* = 7.9990 (16) Å	µ = 3.01 mm^−^^1^
*c* = 10.152 (2) Å	*T* = 293 K
β = 105.88 (3)°	Block, colorless
*V* = 1372.8 (5) Å^3^	0.15 × 0.15 × 0.10 mm
*Z* = 4	

### Data collection

**Table:**

Bruker SMART CCD diffractometer	2684 independent reflections
Radiation source: fine-focus sealed tube	2081 reflections with *I* > 2σ(*I*)
Graphite monochromator	*R*_int_ = 0.034
φ and ω scans	θ_max_ = 26.0°, θ_min_ = 2.4°
Absorption correction: multi-scan (*SADABS*; Bruker, 1997)	*h* = −21→21
*T*_min_ = 0.661, *T*_max_ = 0.753	*k* = −9→9
7399 measured reflections	*l* = −10→12

### Refinement

**Table:**

Refinement on *F*^2^	Primary atom site location: structure-invariant direct methods
Least-squares matrix: full	Secondary atom site location: difference Fourier map
*R*[*F*^2^ > 2σ(*F*^2^)] = 0.036	Hydrogen site location: inferred from neighbouring sites
*wR*(*F*^2^) = 0.086	H atoms treated by a mixture of independent and constrained refinement
*S* = 1.04	*w* = 1/[σ^2^(*F*_o_^2^) + (0.0287*P*)^2^ + 0.3626*P*] where *P* = (*F*_o_^2^ + 2*F*_c_^2^)/3
2684 reflections	(Δ/σ)_max_ = 0.001
187 parameters	Δρ_max_ = 0.36 e Å^−^^3^
2 restraints	Δρ_min_ = −0.38 e Å^−^^3^

### Special details

**Table:**

Geometry. All e.s.d.'s (except the e.s.d. in the dihedral angle between two l.s. planes) are estimated using the full covariance matrix. The cell e.s.d.'s are taken into account individually in the estimation of e.s.d.'s in distances, angles and torsion angles; correlations between e.s.d.'s in cell parameters are only used when they are defined by crystal symmetry. An approximate (isotropic) treatment of cell e.s.d.'s is used for estimating e.s.d.'s involving l.s. planes.
Refinement. Refinement of *F*^2^ against ALL reflections. The weighted *R*-factor *wR* and goodness of fit *S* are based on *F*^2^, conventional *R*-factors *R* are based on *F*, with *F* set to zero for negative *F*^2^. The threshold expression of *F*^2^ > σ(*F*^2^) is used only for calculating *R*-factors(gt) *etc*. and is not relevant to the choice of reflections for refinement. *R*-factors based on *F*^2^ are statistically about twice as large as those based on *F*, and *R*- factors based on ALL data will be even larger.

### Fractional atomic coordinates and isotropic or equivalent isotropic displacement parameters (Å^2^)

**Table:**

	*x*	*y*	*z*	*U*_iso_*/*U*_eq_	
Br1	0.299001 (18)	0.53711 (4)	0.36253 (3)	0.04487 (13)	
N1	0.51901 (17)	0.2174 (4)	0.9902 (3)	0.0556 (7)	
H1A	0.5634 (10)	0.181 (4)	1.041 (3)	0.067*	
H1B	0.506 (2)	0.193 (5)	0.9045 (10)	0.067*	
N2	0.09788 (14)	0.6339 (3)	1.1536 (2)	0.0374 (6)	
N3	0.27770 (14)	0.5158 (3)	0.9346 (2)	0.0333 (5)	
O1	0.48818 (12)	0.3289 (3)	1.1724 (2)	0.0515 (6)	
O2	0.11894 (12)	0.7569 (3)	1.1007 (2)	0.0468 (5)	
O3	0.06762 (15)	0.6449 (3)	1.2487 (3)	0.0648 (7)	
C1	0.47318 (17)	0.3051 (4)	1.0485 (3)	0.0396 (7)	
C2	0.40031 (16)	0.3825 (3)	0.9549 (3)	0.0330 (6)	
C3	0.38718 (18)	0.3982 (4)	0.8142 (3)	0.0411 (7)	
H3	0.4249	0.3592	0.7728	0.049*	
C4	0.31891 (19)	0.4710 (4)	0.7355 (3)	0.0444 (8)	
H4	0.3101	0.4811	0.6412	0.053*	
C5	0.26429 (18)	0.5282 (4)	0.7981 (3)	0.0397 (7)	
H5	0.2175	0.5760	0.7459	0.048*	
C6	0.34414 (16)	0.4456 (3)	1.0136 (3)	0.0329 (6)	
H6	0.3523	0.4396	1.1080	0.040*	
C7	0.21733 (16)	0.5811 (3)	0.9988 (3)	0.0340 (7)	
H7A	0.1859	0.6657	0.9398	0.041*	
H7B	0.2436	0.6335	1.0854	0.041*	
C8	0.16318 (15)	0.4428 (3)	1.0230 (3)	0.0298 (6)	
C9	0.16802 (17)	0.2812 (4)	0.9765 (3)	0.0372 (7)	
H9	0.2030	0.2585	0.9246	0.045*	
C10	0.12235 (18)	0.1532 (4)	1.0052 (3)	0.0443 (8)	
H10	0.1267	0.0464	0.9717	0.053*	
C11	0.07044 (18)	0.1813 (4)	1.0828 (3)	0.0429 (7)	
H11	0.0404	0.0941	1.1028	0.051*	
C12	0.06342 (17)	0.3402 (4)	1.1305 (3)	0.0394 (7)	
H12	0.0284	0.3615	1.1825	0.047*	
C13	0.10931 (15)	0.4683 (3)	1.1001 (3)	0.0307 (6)	

### Atomic displacement parameters (Å^2^)

**Table:**

	*U*^11^	*U*^22^	*U*^33^	*U*^12^	*U*^13^	*U*^23^
Br1	0.0467 (2)	0.0500 (2)	0.0367 (2)	−0.00517 (15)	0.00936 (14)	0.00137 (15)
N1	0.0406 (17)	0.066 (2)	0.0561 (19)	0.0114 (15)	0.0063 (15)	−0.0072 (16)
N2	0.0361 (14)	0.0353 (15)	0.0415 (15)	0.0064 (11)	0.0120 (11)	−0.0022 (12)
N3	0.0364 (14)	0.0322 (13)	0.0346 (13)	−0.0001 (10)	0.0152 (11)	0.0025 (10)
O1	0.0402 (12)	0.0710 (16)	0.0435 (14)	0.0001 (11)	0.0117 (10)	0.0118 (12)
O2	0.0491 (13)	0.0332 (12)	0.0613 (15)	−0.0007 (10)	0.0202 (11)	−0.0023 (11)
O3	0.0894 (19)	0.0559 (16)	0.0689 (16)	0.0129 (13)	0.0549 (15)	−0.0026 (13)
C1	0.0320 (16)	0.0412 (18)	0.047 (2)	−0.0050 (13)	0.0134 (14)	0.0044 (15)
C2	0.0314 (15)	0.0317 (16)	0.0369 (16)	−0.0060 (12)	0.0111 (12)	0.0002 (13)
C3	0.0445 (18)	0.0419 (17)	0.0431 (18)	−0.0009 (14)	0.0224 (15)	−0.0049 (15)
C4	0.053 (2)	0.0526 (19)	0.0301 (16)	0.0042 (16)	0.0158 (14)	0.0022 (15)
C5	0.0418 (17)	0.0398 (17)	0.0348 (17)	0.0044 (14)	0.0056 (14)	0.0070 (14)
C6	0.0343 (15)	0.0359 (17)	0.0289 (15)	−0.0028 (13)	0.0092 (12)	0.0045 (12)
C7	0.0352 (16)	0.0331 (16)	0.0373 (16)	0.0037 (12)	0.0160 (13)	0.0013 (12)
C8	0.0278 (14)	0.0323 (16)	0.0274 (14)	0.0010 (11)	0.0044 (11)	0.0011 (12)
C9	0.0395 (16)	0.0374 (17)	0.0363 (17)	0.0032 (13)	0.0131 (13)	−0.0050 (13)
C10	0.0530 (19)	0.0298 (17)	0.0490 (19)	−0.0039 (14)	0.0121 (15)	−0.0054 (14)
C11	0.0438 (18)	0.0368 (18)	0.0486 (19)	−0.0129 (14)	0.0134 (15)	0.0015 (15)
C12	0.0330 (16)	0.0449 (19)	0.0425 (18)	−0.0027 (13)	0.0141 (13)	0.0028 (14)
C13	0.0307 (14)	0.0283 (14)	0.0320 (15)	−0.0001 (12)	0.0067 (12)	−0.0019 (12)

### Geometric parameters (Å, º)

**Table:**

N1—C1	1.324 (4)	C4—H4	0.9300
N1—H1A	0.860 (2)	C5—H5	0.9300
N1—H1B	0.860 (2)	C6—H6	0.9300
N2—O2	1.226 (3)	C7—C8	1.523 (4)
N2—O3	1.227 (3)	C7—H7A	0.9700
N2—C13	1.466 (4)	C7—H7B	0.9700
N3—C5	1.344 (4)	C8—C9	1.387 (4)
N3—C6	1.345 (4)	C8—C13	1.398 (4)
N3—C7	1.484 (3)	C9—C10	1.381 (4)
O1—C1	1.227 (4)	C9—H9	0.9300
C1—C2	1.503 (4)	C10—C11	1.377 (4)
C2—C6	1.381 (4)	C10—H10	0.9300
C2—C3	1.389 (4)	C11—C12	1.377 (4)
C3—C4	1.376 (4)	C11—H11	0.9300
C3—H3	0.9300	C12—C13	1.390 (4)
C4—C5	1.367 (4)	C12—H12	0.9300
			
C1—N1—H1A	119 (2)	C2—C6—H6	120.0
C1—N1—H1B	124 (3)	N3—C7—C8	111.6 (2)
H1A—N1—H1B	118 (4)	N3—C7—H7A	109.3
O2—N2—O3	122.4 (3)	C8—C7—H7A	109.3
O2—N2—C13	118.3 (2)	N3—C7—H7B	109.3
O3—N2—C13	119.3 (3)	C8—C7—H7B	109.3
C5—N3—C6	121.6 (2)	H7A—C7—H7B	108.0
C5—N3—C7	118.8 (2)	C9—C8—C13	116.1 (2)
C6—N3—C7	119.6 (2)	C9—C8—C7	121.5 (2)
O1—C1—N1	123.7 (3)	C13—C8—C7	122.2 (2)
O1—C1—C2	119.4 (3)	C10—C9—C8	121.8 (3)
N1—C1—C2	116.9 (3)	C10—C9—H9	119.1
C6—C2—C3	118.3 (3)	C8—C9—H9	119.1
C6—C2—C1	117.6 (3)	C11—C10—C9	120.9 (3)
C3—C2—C1	124.1 (3)	C11—C10—H10	119.5
C4—C3—C2	120.6 (3)	C9—C10—H10	119.5
C4—C3—H3	119.7	C10—C11—C12	119.3 (3)
C2—C3—H3	119.7	C10—C11—H11	120.4
C5—C4—C3	118.9 (3)	C12—C11—H11	120.4
C5—C4—H4	120.6	C11—C12—C13	119.3 (3)
C3—C4—H4	120.6	C11—C12—H12	120.4
N3—C5—C4	120.5 (3)	C13—C12—H12	120.4
N3—C5—H5	119.8	C12—C13—C8	122.6 (3)
C4—C5—H5	119.8	C12—C13—N2	115.9 (2)
N3—C6—C2	120.1 (3)	C8—C13—N2	121.4 (2)
N3—C6—H6	120.0		
			
O1—C1—C2—C6	−14.2 (4)	N3—C7—C8—C13	170.4 (2)
N1—C1—C2—C6	167.5 (3)	C13—C8—C9—C10	−0.2 (4)
O1—C1—C2—C3	164.0 (3)	C7—C8—C9—C10	176.1 (3)
N1—C1—C2—C3	−14.3 (4)	C8—C9—C10—C11	−0.6 (5)
C6—C2—C3—C4	−1.7 (4)	C9—C10—C11—C12	0.9 (5)
C1—C2—C3—C4	−179.9 (3)	C10—C11—C12—C13	−0.5 (5)
C2—C3—C4—C5	0.2 (5)	C11—C12—C13—C8	−0.3 (4)
C6—N3—C5—C4	−0.8 (4)	C11—C12—C13—N2	179.5 (3)
C7—N3—C5—C4	179.3 (3)	C9—C8—C13—C12	0.7 (4)
C3—C4—C5—N3	1.0 (5)	C7—C8—C13—C12	−175.6 (3)
C5—N3—C6—C2	−0.7 (4)	C9—C8—C13—N2	−179.2 (2)
C7—N3—C6—C2	179.2 (2)	C7—C8—C13—N2	4.6 (4)
C3—C2—C6—N3	1.9 (4)	O2—N2—C13—C12	−160.2 (3)
C1—C2—C6—N3	−179.8 (2)	O3—N2—C13—C12	19.6 (4)
C5—N3—C7—C8	97.2 (3)	O2—N2—C13—C8	19.7 (4)
C6—N3—C7—C8	−82.7 (3)	O3—N2—C13—C8	−160.5 (3)
N3—C7—C8—C9	−5.6 (4)		

### Hydrogen-bond geometry (Å, º)

**Table:**

*D*—H···*A*	*D*—H	H···*A*	*D*···*A*	*D*—H···*A*
N1—H1*B*···O1^i^	0.86 (1)	2.30 (1)	3.143 (4)	168 (4)
N1—H1*A*···Br1^ii^	0.86 (1)	2.61 (1)	3.454 (3)	166 (3)
C4—H4···Br1	0.93	2.82	3.743 (3)	173
C7—H7*B*···Br1^iii^	0.97	2.82	3.595 (3)	137
C5—H5···O2^iv^	0.93	2.36	3.271 (4)	167
C3—H3···O1^i^	0.93	2.27	3.150 (4)	157

Symmetry codes: (i) *x*, −*y*+1/2, *z*−1/2; (ii) −*x*+1, *y*−1/2, −*z*+3/2; (iii) *x*, *y*, *z*+1; (iv) *x*, −*y*+3/2, *z*−1/2.
